# Immunodiagnosis of cystic echinococcosis in livestock: Development and validation dataset of an ELISA test using a recombinant B8/2 subunit of *Echinococcus granulosus sensu lato*

**DOI:** 10.1016/j.dib.2022.108255

**Published:** 2022-05-11

**Authors:** Thelma Verónica Poggio, José Manuel Gómez, Lorena Analia Boado, Adrián Alberto Vojnov, Edmundo Larrieu, Guillermo B. Mujica, Oscar Jensen, Maria Laura Gertiser, Joaquin M. Prada, Maria-Gloria Basáñez

**Affiliations:** a Laboratorio de Diseño y Desarrollo, Instituto de Ciencia y Tecnología César Milstein (ICT-Milstein-CONICET), Buenos Aires, Argentina; b Facultad de Ciencias Veterinarias, Universidad Nacional de La Pampa, General Pico, Argentina; c Escuela de Veterinaria, Universidad Nacional de Río Negro, Choele Choel, Argentina; d Ministerio de Salud, Provincia de Río Negro, Viedma, Argentina; e Centro de Investigación en Zoonosis, Provincia de Chubut, Argentina; f Faculty of Health and Medical Sciences, University of Surrey, Guildford, UK; g London Centre for Neglected Tropical Disease Research and MRC Centre for Global Infectious Disease Analysis, Faculty of Medicine, School of Public Health, Imperial College London, London, UK

**Keywords:** Cystic echinococcosis, Serodiagnosis, ELISA, Sheep, Recombinant AgB8/2, *Echinococcus granulosus sensu lato*, CE, Cystic echinococcosis, ELISA, Enzyme-linked immunosorbent assay, OD, Optical density, *s.l.*, sensu lato

## Abstract

The accuracy of screening tests for detecting cystic echinococcosis (CE) in livestock depends on characteristics of the host–parasite interaction and the extent of serological cross-reactivity with other taeniid species. The AgB8 kDa protein is considered to be the most specific native or recombinant antigen for immunodiagnosis of ovine CE. A particular DNA fragment coding for rAgB8/2 was identified, that provides evidence of specific reaction in the serodiagnosis of metacestode infection.

We developed and validated an IgG Enzyme Linked Immunosorbent Assay (ELISA) test using a recombinant antigen B sub-unit EgAgB8/2 (rAgB8/2) of *Echinoccocus granulosus sensu lato* (*s.l.*) to estimate CE prevalence in sheep. A 273 bp DNA fragment coding for rAgB8/2 was expressed as a fusion protein (∼30 kDa) and purified by affinity chromatography.

Evaluation of the analytical and diagnostic performance of the ELISA followed the World Organisation for Animal Health (OIE) manual, including implementation of serum panels from: uninfected lambs (*n* = 79); experimentally infected (with 2,000 *E. granulosus s.l.* eggs each) sheep with subsequent evidence of *E. granulosus* cysts by necropsy (*n* = 36), and animals carrying other metacestode/trematode infections (*n* = 20). The latter were used to assess the cross-reactivity of rAgB8/2, with these animals being naturally infected with *Taenia hydatigena, Thysanosoma actinioides and/or Fasciola hepatica*. EgAgB8/2 showed cross-reaction with only one serum sample from a sheep infected with *Ta. hydatigena* out of the 20 animals tested.

Furthermore, the kinetics of the humoral response over time in five 6-month old sheep, each experimentally infected with 2,000 *E. granulosus s.l.* eggs, was evaluated up to 49 weeks (approximately one year) post infection (*n* = 5). The earliest detectable IgG response against rAgB8/2 was observed in sera from two and four sheep, 7 and 14 days after experimental infection, respectively. The highest immune response across all five animals was found 16 to 24 weeks post infection.

## Specifications Table

**Table:** 

Subject	Biological sciences – Parasitology
Specific subject area	Immunodiagnostics; recombinant antigen design and evaluation; serodiagnosis of ovine cestodiasis for cystic echinococcosis control and surveillance
Type of data	.csv Files (https://github.com/joaquinprada/CE-ELISA-recombinant) Figures (Figure 1) Graphs (Figure 2, Figure 3)
How the data were acquired	Sheep serum samples were generated from blood samples collected by jugular venipuncture through standard, ethically approved, and aseptic procedures. Serum samples were analysed by indirect ELISA. Necropsy data were collected on site through visual inspection, palpation and slicing of organs (e.g. liver, lungs). More details are described below.
Data format	Raw (see .csv Files with raw data Tables) Analysed (Figures 1–3)
Description of data collection	Negative Serum Panel: Lambs aged ≤3 months from a CE-free area (*n* = 79). Positive Serum Panel: Experimentally-infected, necropsy-positive sheep (with *E. granulosus* cysts in internal organs) (*n* = 36). Cross-Reactivity Sera: Sheep naturally infected with other cestodes/trematodes (*n* = 20). Humoral Response Kinetics: Sera from CE experimentally-infected sheep evaluated for up to 49 weeks post infection (*n* = 5).
Data source location	Serum Negative Panel: • *City/Town/Region:* Puerto Madryn, 42.7636° S, 65.0348° W (Chubut Province) • *Country:* Argentina Serum Positive Panel and Sera from CE experimentally-infected sheep: • *Institution:* Centro de Investigación en Zoonosis, 45.6667° S, 69.0833° W • *City/Town/Region:* Sarmiento, Chubut Province • *Country:* Argentina Sera from sheep naturally infected with other cestodes and/or trematodes: • *City/Town/Region:* Río Negro Province, 41.3215° S, 70.2747° W • *Country:* Argentina
Data accessibility	Repository name: GitHub Title of the dataset: Dataset of an ELISA test using recombinant B8/2 subunit for *Echinococcus granulosus sensu lato* Direct URL to data: https://github.com/joaquinprada/CE-ELISA-recombinant
Related research article	A.L. Sykes, E. Larrieu, T.V. Poggio, M.G. Céspedes, G.B. Mujica, M.G. Basáñez, J.M. Prada, Modelling diagnostics for *Echinococcus granulosus* surveillance in sheep using Latent Class Analysis: Argentina as a case study, One Health. 14 (2021) 100359, 10.1016/j.onehlt.2021.100359[1].

## Value of the data

• *Importance:* We identified a particular DNA fragment coding for rAgB8/2 that provides evidence of specific reaction for the serodiagnosis of *Echinococcus granulosus s.l.* metacestode infection.
• *Novelty:* The rEgAgB8/2 antigen had been demonstrated to be an appropriate antigen for serological diagnosis of human CE [2], but not previously applied for CE diagnosis in sheep.
• *Programmatic applications:*

*Usefulness for CE control and surveillance:* The rEgAgB8/2 indirect ELISA for ovine CE developed and reported here would be particularly useful for flock diagnosis in control and surveillance programmes [1].
*Prevention of CE infection introduction or re-introduction:* The ELISA test described here could be used in serological surveys to detect cyst-bearing animals from endemic areas in transit to CE-free areas to minimise propagation of *E. granulosus s.l.*, as well as to identify farms that may represent potential sources of infection or re-infection to CE-controlled farms.
*Better targeting of CE interventions:* The application of ELISA serology using the data described would help identify sheep farms in which interventions (e.g. sheep vaccination) would need to be deployed or intensified.

• *Diagnostic applications:*

*Assessment of ELISA diagnostic performance:* The data presented here would be useful for evaluation of the diagnostic performance of the ELISA in (within- and between-country) inter-laboratory comparisons, in which different operators, equipment, reagents and temperature conditions may vary. Determination of its repeatability and reproducibility will be essential for its validation and adoption in CE control and surveillance programmes.

• *Epidemiological applications:*

*Quantification of ovine CE prevalence:* Data generated by applying the ELISA test presented here can be used in conjunction with other diagnostics to improve estimates of ovine CE prevalence [1] as necropsy is only a partial gold standard which can miss early/small-size cyst infections [3]. Its use for flock-level diagnosis can help improve CE surveillance [4].
*Quantification of ovine CE incidence:* The temporal kinetics of the humoral response against AgB8/2 following CE experimental infection of sheep will provide important information for interpreting force-of-infection (the per susceptible rate of infection acquisition) modelling studies using age-stratified seroprevalence data.
*Quantification of disease burden:* This diagnostic tool would facilitate quantification of ovine disease burden and of progress towards CE control in areas with long-standing programmes where infection by *E. granulosus s.l*. remains a public health problem.

## 1 Data Description

All data were gathered through the analysis of serum samples collected by control and surveillance programmes conducted in Río Negro and Chubut Provinces, and laboratory work performed at the Centro de Investigación en Zoonosis, Sarmiento, Chubut, Argentina. All data were entered on a database and analysed.

**Fig. 1:**
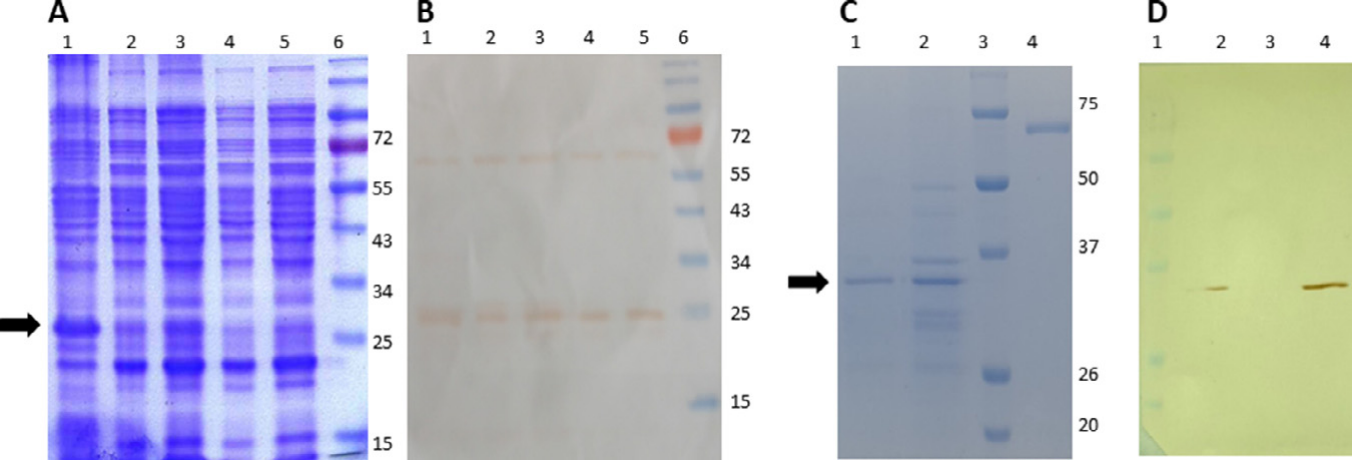
Expression and purification of rAgB8/2. **(A)** SDS-PAGE 12%: lane 1, EgAgB8/2 solubilized as inclusion bodies (IB) in 8 M Urea, 0.5 M ClNa, PBS pH 7.2; lane 2, IB pellet before induction; lane 3, IB pellet after induction; lanes 4–5, Supernatant proteins after induction; lane 6, Molecular weight (MW) marker (kDa). **(B)** Western blot: lanes 1–5, Immunological characterization of rEgAgB8/2 protein in different fractions using anti-histidine (His)-tag monoclonal antibody (mAb); lane 6, MW marker (kDa). **(C)** Coomassie blue-stained gel 12%: lane 1, purification of His-tagged rAgB/2 protein with (nitrilotriacetic acid) Ni-NTA affinity chromatography; lane 2, EgAgB8/2 solubilized as IB diluted; lane 3, MW marker (kDa); lane 4, Bovine serum albumin (BSA) as negative control. **(D)** Western blot: lane 1, MW marker (kDa); lane 2, Immunological characterization of purified rAgB8/2 probed with anti-His-tag mAb; lane 3, BSA as negative control; lane 4, purified rAgB8/2 probed with serum from *E. granulosus*-infected sheep. Horizontal black arrows indicate target bands specific to the recombinant protein.

**Fig. 2:**
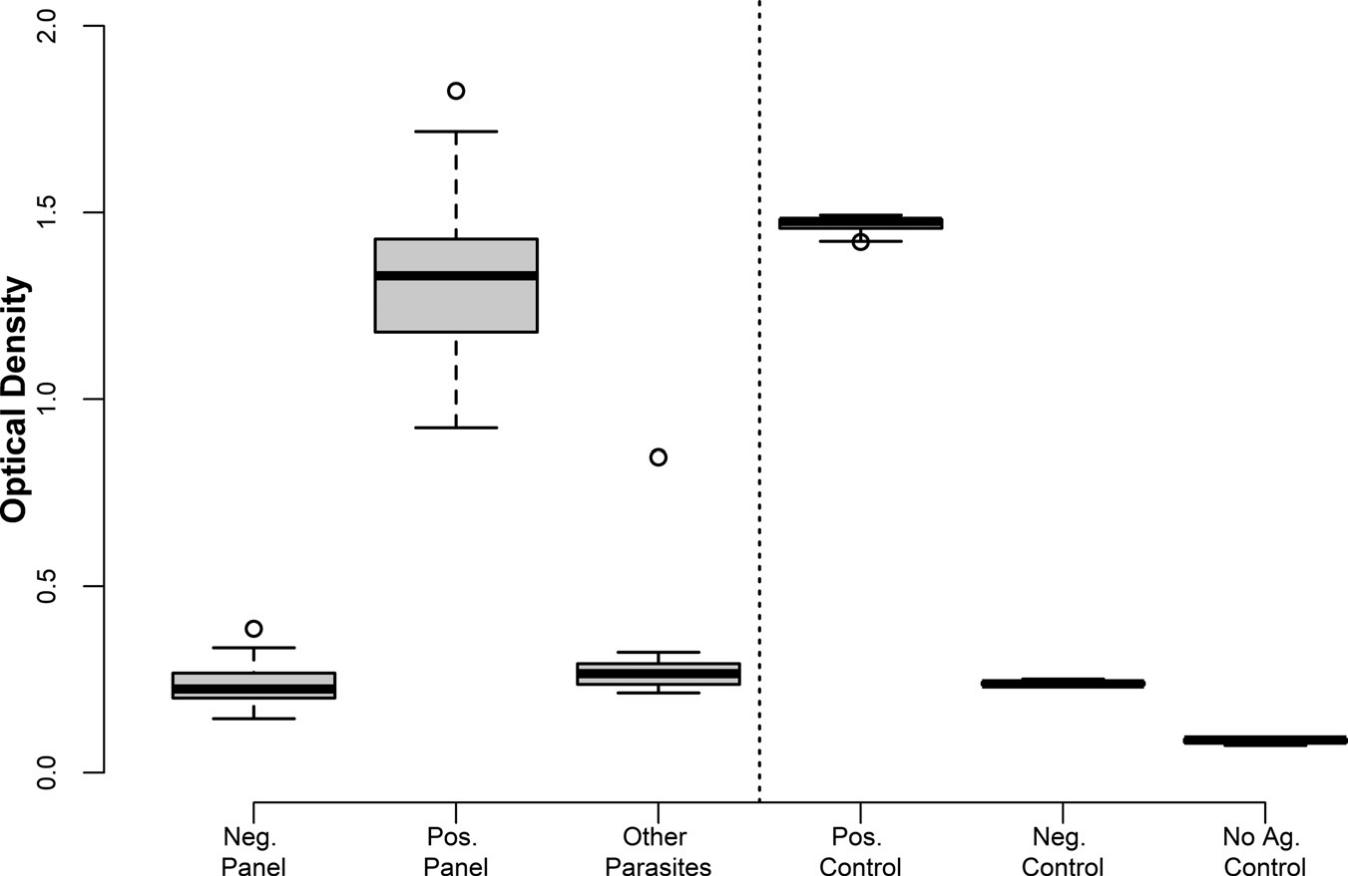
Box plots of IgG Optical Density (OD)_405_ _nm_ from sheep sera: Negative Serum Panel (*n* = 79), lambs under 3 months of age from a CE-free area; Positive Serum Panel (*n* = 36), adults experimentally infected with positive necropsy; Cross-reactive Sera (*n* = 20), animals with other parasites. The solid black horizontal line within the boxes is the median; the lower and upper borders are, respectively, the 1st (Q1) and 3rd (Q3) quartiles; the vertical bars indicate the ‘minimum’ and ‘maximum’ values, calculated as Q1–1.5 × IQR (interquartile range) and Q1+1.5 × IQR, respectively. The circles represent the outliers. Laboratory (positive, negative and no-antigen) controls are presented to the right of the vertical dotted line.

**Fig. 3:**
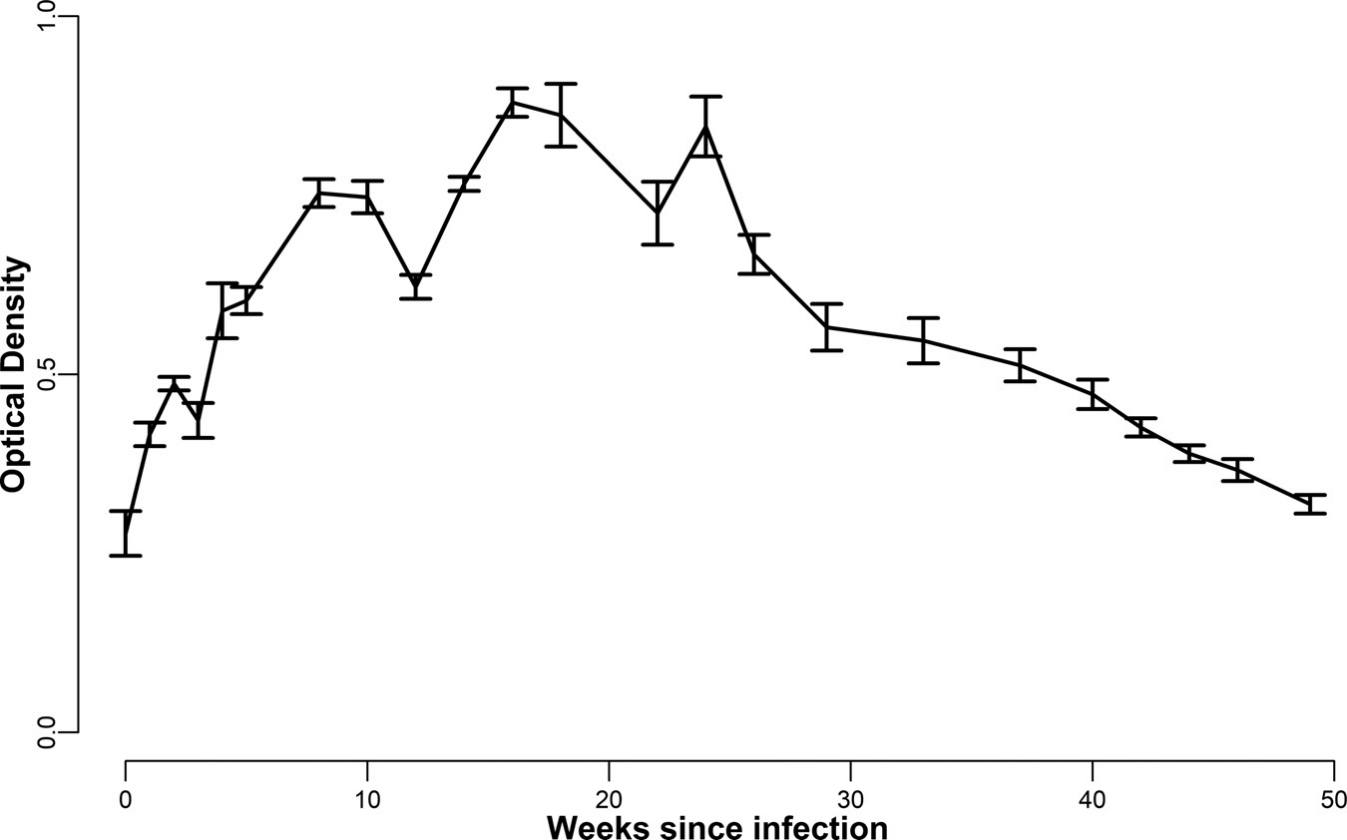
Average Optical Density (OD)_405_ _nm_ of antibody levels against rAg8/2 in serum samples from sheep experimentally infected with 2000 *E. granulosus s.l.* eggs (*n* = 5) plotted against time post infection over 49 weeks. The error bars are the standard errors of the means (SEM).

Datasets: .csv files containing the raw data generated and used in the figures described below (https://github.com/joaquinprada/CE-ELISA-recombinant).

## 2 Experimental Design, Materials and Methods

### 2.1 Recombinat B8/2 antigen B subunit ELISA for *E. granulosus s.l.* detection

**Gene optimization, cloning and expression:** Sequence data were obtained from NCBI GenBank with accession number AY569349. Gene optimization of AgB8/2 for heterologous expression in *Escherichia coli* (including sites BamH I and EcoR I) was conducted by cloning in pGEX-1λT (Sigma-Aldrich) vector, and plasmid transformation into BL21 (DE3) cells for expression under the T7 promoter was performed. rAgB8/2 was expressed in fusion with N-terminal GST and C-terminal His-tag in BL21(DE3) *E. coli strain* by 0.5 mM IPTG (Invitrogen, USA) induction for 4 h. The produced *E. granulosus s.l.* recombinant AgB8/2 antigen (rEgAgB8/2) was solubilized as an inclusion body in 8 M Urea, 0.5 M ClNa, PBS pH 7.2 and stirred overnight at 4 °C. The antigen was subsequently diluted to 2 M Urea, 0.5 M ClNa, PBS pH 7.2, centrifuged at 10,000 rpm for 30 min, and was purified with Protino Ni-TED/IDA (QIAGEN Hilden, Germany) according to the manufacturer's instructions. In brief, 25 ml of solubilised inclusion bodies in 2 M Urea start buffer was added to 1 ml of resin slurry. rAgB8/2 protein plus resin was stirred overnight at 4 °C followed by 2.5 h at room temperature. The resin was washed 3 times in 2 M Urea start buffer/ 20 mM imidazole, once in 2 M urea start buffer/40 mM imidazole and once in start buffer/ 20 mM imidazole. Then, the resin was washed once in start buffer/ 0.1% SDS/ 20 mM imidazole, rotating for 30 min at room temperature. Finally, elution of rAgB8/2 protein with start buffer/0.1% SDS/ 200 mM imidazole was carried out. Sodium dodecyl sulphate-Polyacrylamide gel electrophoresis (SDS-PAGE) and Western blot analyses of the recombinant protein were evaluated by use of the pooled sera from *E. granulosus* experimentally-infected and necropsy-positive sheep (positive control), and *E. granulosus*-negative lambs (negative control).

### 2.2 Collection of sheep sera

Serum samples from lambs and sheep were obtained from jugular venipuncture to evaluate the specific antibody response against rAgB8/2 by ELISA. Evaluation of the analytical and diagnostic performance of the ELISA followed the World Organisation for Animal Health (OIE) manual [5]:

**Negative Serum Panel:** Sera collected from healthy Merino lambs aged under 3 months, belonging to the non-endemic area of Puerto Madryn (Chubut Province, Argentina) (*n* = 79).
**Positive Serum Panel:** Adult sheep from the Chubut Province, each experimentally infected *per os* with 2,000 *E. granulosus s.l.* eggs in May 2013, were necropsied two years later in 2015. Live cysts (from 2 to 249) larger than 10 mm were detected in all animals, demonstrating *E. granulosus s.l.* infection (*n* = 36).
**Cross-Reactive Sera:** Sera from sheep naturally infected with *Taenia hydatigena, Thysanosoma actinioides* and *Fasciola hepatica* were collected from Río Negro Province (*n* = 20) for investigation of cross-reactivity of the IgG rAg8/2 ELISA.
**Humoral Response Kinetics:** Six-month old female sheep (*n* = 5) were each experimentally infected *per os* with 2,000 *E. granulosus s.l.* eggs. Sera were collected by obtaining blood samples every 7 to 15 days for 49 weeks for investigation of the temporal kinetics of IgG antibody response to the rAg8/2 antigen measured as optical density (OD) at 405 nm.
**Preparation of *E. granulosus s.l.* Eggs for Experimental Infections:** Eggs originated from mature worms obtained from 30 naturally infected farm dogs dewormed with arecoline hydrobromide [6], [7], [8] in Trevelin, Departament of Futaleufú, Province of Chubut (Río Frío farm, 43.4333° S, 71.6333° W, Altitude/elevation: 704 m (2309 ft).

All serum samples were stored at ICT-Milstein-CONICET at –20 °C until used.

### 2.3 IgG ELISA using rAgB8/2

The rAgB8/2 antigen was kept at –20 °C and diluted 1 : 400 with carbonate/bicarbonate coating buffer pH 9.0 (Sigma-Aldrich Corp. St. Louis, MO, USA) for coating Nunc-Immuno™ MicroWell™ 96-well solid plates (Sigma-Aldrich) (50 µL/well). The coating buffer was discarded and the plates were washed three times, for 3 min per wash, with washing buffer (0.15 M phosphate-buffered saline containing 0.05% Tween 20 (Sigma)). Plates were then blocked with 200 µL/well of blocking solution (900 mL phosphate-buffered saline, 100 mL adult horse serum, 1% phenol red) for 1 hour at room temperature. The blocking buffer was discarded, and the plates were washed three times. Then, 100 µL of (positive and negative) control sera, with dilutions ranging from 200 to 25,600 in blocking solution, was added to each well and plates were incubated at room temperature for 90 min. Plates were then washed three times and 100 µL of donkey anti-sheep IgG-HRP (horseradish peroxidase) conjugate 1 : 3000 (Invitrogen, Carlsbad, CA, USA) in blocking solution was added and plates were left for 1 h at room temperature. Plates were washed three times and 100 µL of ABTS (2,20-azino-bis(3-ethylbenzothiazoline-6-sulphonic acid), 0.5 mg/mL in 70 mM citrate phosphate buffer, pH 4.2) with 8 µL of 30% hydrogen peroxide per 6 mL of substrate was added to each well immediately prior to use. Plates were incubated in the dark for 20 min. When the first hint of colour showed in the negative controls, the reaction was stopped by the addition of 50 µL of 2% sodium fluoride to each well. Plates were then read at 405 nm using an automated ELISA plate reader. The negative control of each assay was titrated with the positive control on the same plate and provided a visual endpoint for stopping the development of the colour reaction. Standard curves were generated for each ELISA plate using the absorbance values of the positive control, in comparison with the negative control, for dilutions ranging from 1 : 200 to 1 : 25,600. The conditions of acceptance of each plate were as follows: the negative control optical density (OD) should be ≤0.400; the positive control OD should be >0.750 but <1.950; the OD of the positive control should be 3 times higher than the OD of the negative control. The cut-off value should be 2 times the mean OD of the negative control plus 2 times the SD around this mean and was determined as 0.492.

### 2.4 Necropsy

Liver, heart, spleen, lungs and kidneys of each animal were finely sliced (3 mm slices for liver and 5 mm slices for lungs), and each slice was inspected and palpated to find any cysts. All cysts were sliced through the middle with a sharp scalpel to confirm that they were *E. granulosus s.l.* cysts, either with a fluid-filled central cavity indicating viability, or a caseous centre with no cavity, indicating death during early development.

## Ethics Statement

All experiments complied with the ARRIVE (Animal Research: Reporting of In Vivo Experiments) guidelines and were carried out in accordance with the U.K. Animals (Scientific Procedures) Act, 1986 and associated guidelines; EU Directive 2010/63/EU for animal experiments and the National Institutes of Health guide for the care and use of laboratory animals (NIH Publications No. 8023, revised 1978).

## CRediT authorship contribution statement

**Thelma Verónica Poggio:** Conceptualization, Validation, Formal analysis, Writing – original draft. **José Manuel Gómez:** Methodology, Investigation, Visualization, Validation. **Lorena Analia Boado:** Methodology, Investigation, Visualization, Validation. **Adrián Alberto Vojnov:** Methodology, Investigation, Visualization, Validation, Resources, Project administration. **Edmundo Larrieu:** Resources, Project administration, Formal analysis, Supervision, Writing – review & editing. **Guillermo B. Mujica:** Investigation. **Oscar Jensen:** Investigation, Resources, Project administration. **Maria Laura Gertiser:** Investigation, Resources. **Joaquin M. Prada:** Visualization, Writing – review & editing. **Maria-Gloria Basáñez:** Visualization, Writing – review & editing.

## Declaration of Competing Interest

The authors declare that they have no known competing financial interests or personal relationships that could have appeared to influence the work reported in this paper. The funders had no role in the study design, data collection and analysis, decision to publish, or preparation of the manuscript.

## Data Availability

Dataset of an ELISA test using a recombinant B8/2 subunit of Echinococcus granulosus sensu lato (Original data) (https://github.com/joaquinprada/CE-ELISA-recombinant).
